# miR-6086 inhibits ovarian cancer angiogenesis by downregulating the OC2/VEGFA/EGFL6 axis

**DOI:** 10.1038/s41419-020-2501-5

**Published:** 2020-05-11

**Authors:** Binhua Wu, Ligang Zhang, Yunfei Yu, Tongyi Lu, Yinmei Zhang, Wenhui Zhu, Qifang Song, Chengding Lv, Jiaying Guo, Yiqiao Tian, Ning Deng

**Affiliations:** Guangdong Province Engineering Research Center for Antibody Drug and Immunoassay, Department of Biology, Jinan University, Guangzhou, 510632 China

**Keywords:** Targeted therapies, Tumour angiogenesis

## Abstract

miRNAs have emerged as a pivotal component of gene regulatory networks, mediating cytokines secretion, cell cycle, and differentiation regulation. However, how miRNAs collaborate with transcription factors and downstream effector proteins that determine the fate of ovarian cancer cells remains to be understood, especially regarding to mechanism of tumor angiogenesis regulation. Based on the qRT-PCR and IHC analysis, we found that miR-6086 was maintained a very low level both in ovarian cancer cell lines and tissues. Further, we identified OC2 and EGFL6 as the direct targets of miR-6086 by luciferase assay and we observed an inverse relationship between the expression of miR-6086 and the OC2/VEGFA/EGFL6 axis. The Western blotting analysis suggested that OC2 could directly upregulate VEGFA and indirectly up-regulate EGFL6 through VEGFA. Moreover, miR-6086 could indirectly downregulate VEGFA through OC2. In addition, miR-6086, siOC2 and siEGFL6 could negatively regulate the tumor growth and angiogenesis of ovarian cancer (Skov3) in the animal studies, with the inhibition rates of 77.07%, 69.89%, and 73.62%, respectively (**p < 0.01). Moreover, the tumor cell proliferation, migration, and invasion of ovarian cancer cell lines (Caov3 and Skov3) and vascular formation (HUVECs) were significantly suppressed in vitro, by decreasing the AKT/MAPK pathways (*p < 0.05). Taken together, our results reveal that miR-6086 can suppress the angiogenesis networks in ovarian cancer by down-regulating the OC2/VEGFA/EGFL6 axis, directly or indirectly, which may provide potential targets for tumor therapeutics.

## Introduction

Ovarian cancer is one of the leading causes of death among female genital malignancies with high mortality, high recurrence rate, and low survival outcome^1^. Therapy of this malignant tumor suffers from lack of effective strategies, clinical heterogeneity, and poor prognosis in patients^2^. Meanwhile, angiogenesis is an essential program for ovarian cancer, which is induced by numerous angiogenic factors, such as vascular endothelial growth factor A (VEGFA), fibroblast growth factor (FGF2), platelet-derived growth factor subunit A (PDGFA), EGF-like domain multiple 6 (EGFL6), and so on^3–6^. Therefore, direct interference targeting these angiogenic factors can effectively suppress angiogenesis and tumor development^7^. However, current antiangiogenic strategies for cancer, such as the inhibition of growth factors, receptors and kinases, are barely based on single pathway and resulted in modest and transient benefits^8,9^.

MicroRNAs (miRNAs) have been shown to play key roles in angiogenesis and their deregulation has a global impact on tumor angiogenesis networks, offering newer opportunities for cancer therapy^10^. These are endogenous small noncoding RNAs that act as post-transcriptional regulators of gene expression by binding the 3′untranslated region (3′UTR) of target transcripts, leading to translational repression or degradation of mRNA^11–13^. Since single miRNA may regulate gene expression at multiple levels including transcription factors and downstream effector proteins, targeting the activity of miRNAs may be promising^14–16^. Several miRNAs have been shown to negatively regulate oncogenes or tumor-suppressors in tumorigenesis and angiogenesis and thus promoting or suppressing these processes^17–21^. We focused on identifying suppressive miRNAs and their downstream targets in ovarian cancer. miR-6086 is the first reported in 2012 and shows variable expression in diseases^22^. It has been found to be over-expressed post infection with influenza H7N9, while it is downregulated in certain tumor types^23^. Moreover, miR-6086 resides in the EGFL6 gene that mediates migration of endothelial cells via activation of the ERK pathway^24^. Yoo (2012) introduced that the levels of miR-6086 in human umbilical vein endothelial cells (HUVECs) and endothelial cells derived from human embryonic stem cells were significantly decreased and CDH5 was identified as the downstream target of miR-6086^22^. Further, the intronic miRNAs are functionally correlated with their host genes and we suppose that miR-6086 may act as an upstream antiangiogenic regulator of EGFL6^25,26^. However, the action mode of miR-6086 in tumor angiogenesis remains to be fully understood and any unknown mechanisms of vessel formation and regulation networks need further exploration.

In this study, we found that miR-6086 was maintained low level both in ovarian cancer cell lines and tissues, which mediated tumor growth, migration, invasion, and angiogenesis. Next, our analysis identified that OC2 and EGFL6 were the direct targets of miR-6086 and we further demonstrated the relationship between miR-6086 and the OC2/VEGFA/EGFL6 axis in ovarian cancer. Our results offered a new understanding of the role of miR-6086 in regulating angiogenic factors and tumor angiogenesis, which may help us reveal the mechanism of miR-6086 and angiogenesis networks in ovarian cancer for reference in the future.

## Materials and methods

### Tissue samples of ovarian cancer

This study was approved by the ethics committee of the First Affiliated Hospital of Jinan University (Guangzhou, China) and informed consent of all patients were obtained. We obtained 33 malignant and 6 normal ovary tissue samples from the First Affiliated Hospital of Jinan University. The malignant tissues were comprised of malignant adenocarcinoma, mucinous carcinoma, epithelial carcinoma, and mixed tumors.

### Cell culture

The ovarian cancer cell lines Caov3 and ES-2 were purchased from the Shanghai Institute of Biochemistry and Cell Biology, Chinese Academy of Sciences. The ovarian cancer cell lines Cov362, Cov504, EFO-27, OV-90, Skov3, Tov-21G, HUVECs, and HEK293T cells were available in our laboratory. All the cells were cultured in Dulbecco’s modified Eagle’s medium (DMEM, Invitrogen, New York, NY, USA) supplemented with 10% fetal bovine serum (FBS, Hyclone, Logan, UT, USA) and 100 U/mL penicillin/streptomycin (Gibco, Langley, OK, USA) in a 5% CO_2_ incubator at 37 °C.

### Vector constructs

To construct recombinant plasmid of pGCMV-EGFP-miR-6086, the miR-6086 sequence was inserted into pGCMV-EGFP-miR-Blasticidin vector (Genepharma, Shanghai, China). To construct recombinant plasmids of pGPU6-GFP-Neo-OC2-siOC2#1/siOC2#2, pGPU6-GFP-Neo-siEGFL6 and pGPU6-GFP-Neo-siVEGFA, the siRNAs against OC2, EGFL6, and VEGFA were inserted into the pGPU6-GFP-Neo vector (Genepharma). The miR-6086 inhibitor was synthesized by Genepharma. The sequences of miR-6086, siOC2s, siEGFL6, siVEGFA, and negative control have been listed in Supplementary Table 1.

### Transfection

The ViaFect™ transfection reagent (Promega, Madison, WI, USA) was used for transfecting miR-6086 and siRNAs (3 μg/well) into the ovarian cancer cell lines (Caov3 and Skov3) maintained in basal DMEM, following the manufacturer’s instructions. After 6 h, the cells were washed, maintained in DMEM supplemented with 10% FBS for 24 h and collected for subsequent experiments.

### Luciferase assay

The binding sites of miR-6086 in the 3′UTRs of OC2 and EGFL6 were predicted using Targetscan (http://www.targetscan.org/), Pictar (http://pictar.mdc-berlin.de/) and miRDB (http://mirdb.org/miRDB/). Then, the 3′UTR sequences of OC2 and EGFL6 were mutated using the Site-Directed Gene Mutagenesis Kit (Beyotime Biotechnology, Jiangsu, China). To construct pmirGLO-OC2/EGFL6-3′UTR vectors, the 3′UTRs of OC2 and EGLF6 containing the predicted binding sites for miR-6086 were cloned into pmirGLO vector (Promega). Further, the mutated plasmids were similarly constructed. The wild type (WT) and mutated (Mut) were co-transfected with miR-6086 into HEK293T as previously described. Luciferase activity was detected using the Dual-Luciferase Reporter Assay System (Promega) and the values were normalized against Renilla luciferase activity^22^.

### Real-time quantitative polymerase chain reaction (qRT-PCR)

The TRIzol reagent (Life Technologies, Shanghai, China) was used to extract total RNA from the ovarian cancer cells and tissues and the Fermentas K1622 Kit (Fermentas, Burlington, ON, Canada) was subjected to reverse transcription, both following the manufacturer’s instructions. Further, qRT-PCR procedures were performed using the SYBR-Green Master PCR Mix Kit (TAKARA, Shiga, Japan) through 7500 Fast Real-Time PCR System (Applied Biosystems, Foster City, CA, USA). U6 or GAPDH served as the control and the data were analyzed by the 2^−ΔΔCT^ method. The primer sequences, reaction parameters and temperature protocols for miR-6086 and mRNAs detection have been listed in Supplementary Tables 2–4.

### Western blotting assay

Briefly, total protein of ovarian cancer cell lines was extracted using the cell lysis buffer supplemented with protein inhibitor, PMSF, phosphatase inhibitor (Beyotime Biotechnology), and the protein concentration was determined by BCA kit (Thermo Scientific, San Jose, CA, USA). The cell lysates were concentrated with cell culture media only for VEGFA and EGFL6 analysis. An equal amount of sample (30 μg protein) was separated on sodium dodecyl sulfate-polyacrylamide gel electrophoresis and transferred to polyvinylidene fluoride membranes (Millipore, Bedford, MA, USA). After 1-h 5% nonfat milk blocking, the membranes were incubated with primary antibodies overnight at 4 °C, followed by 45-min secondary antibodies incubation at 37 °C. The antibodies of t/p-AKT (Cat: 4691, 1:1000, Cat: 4060, 1:2000), t/p-MAPK (Cat: 4695, 1:1000, Cat: 4370, 1:2000), and GAPDH (Cat: 5174, 1:1000) were from Cell Signaling Technology, Beverly, MA, USA. The antibodies of VEGFA (Cat: ab52917, 1:10,000), OC2 (Cat: ab28466, 1.25 µg/mL) and EGFL6 (Cat: ab140079, 1:1000) were from Abcam, Cambridge, MA, USA. The protein bands were detected by ECL Western Blot Substrate (Millipore) and Gel Documentation System (Bio-rad, Hercules, CA, USA). GAPDH served as loading control and the signal intensity was analyzed by ImageJ software.

### Cell viability

The Caov3 and Skov3 (2.5 × 10^3^ cells/well) were added into the 96-well plates and transfected with miR-6086, siOC2 and siEGFL6, respectively. The CCK-8 solution (10 μL, Dojindo, Kumamoto, Japan) was added to each well in the dark and the absorbance was read at 450 nm using a microplate reader (BioTek Instruments, Winooski, VT, USA).

### Wound-healing assay

The Caov3 and Skov3 (5.0 × 10^5^ cells/well) were transferred to the 6-well plates and transfected with miR-6086, siOC2, and siEGFL6. The monolayer cells were scraped in a straight line by a 200 μL pipette tip and the wounds were washed with basal DMEM to remove the detached cells. The scratched cells were incubated with DMEM supplemented with 0.5% FBS and the photographs were taken at 0 and 24 h under an inverted microscope (Olympus, Tokyo, Japan). The blank areas in the defined site of each scratch were measured by ImageJ software and the migration rates were calculated through dividing the width of wound at 0 and 24 h.

### Cell migration and invasion assays

The cell migration and invasion of Caov3 and Skov3 transfected with miR-6086, siOC2, and siEGFL6 were evaluated using a Transwell-Matrigel system (BD Bioscience, San Jose, CA, USA) in the 24-well plates. For the migration assay, the cells (2 × 10^4^ cells/well) suspended in 150 µL basal DMEM were seeded into the upper chamber and 750 μL DMEM supplemented with 10% FBS was added into the lower chamber. After 24 h incubation, the migrated cells were stained and imaged under the inverted microscope. A similar method was followed for performing the invasion assay, except that the inner membrane of chamber was coated with 50 μL Matrigel. The cell numbers were counted in five random fields for evaluating the cell migration and invasion abilities by ImageJ software.

### Endothelial cell tube formation assay

The HUVECs (1.5 × 10^4^ cells/well) were transferred to 96-well plates coated with Matrigel and incubated for 6 h, with the supernatants from Caov3 and Skov3 transfected with miR-6086, siOC2, and siEGFL6. The cells were imaged under the inverted microscope and the number of loops formed was counted in five random fields and assayed by ImageJ software.

### Mice and tumor model

The BALB/c nude mice (female, 6 weeks) were purchased from the Laboratory Animal Center of Sun Yat-sen University in Guangzhou, China. The Skov3 transfected with miR-6086, siOC2#1, and siEGFL6 were mixed with Matrigel and DMEM (vol/vol, 1:2). The mice were inoculated subcutaneously with these cells (1 × 10^6^ cells/mouse) in the back. Tumor growth was measured in two dimensions using a Caliper (Formula: *π* × length × width^2^/6) and the tumor volume measured at different time points were subjected to mixed-effects model for repeated-measures ANOVA. The animal studies were performed according to the Guidelines of the Institutional Animal Care and Use Committee of Jinan University, Guangzhou, China.

### Immunohistochemistry (IHC)

Tumor tissues from the challenged mice were fixed in 4% paraformaldehyde and embedded in paraffin. After deparaffinization, the sections were subjected to 10 mM sodium citrate buffer (pH 6.0) at 100 °C for 10 min and treated with 3% H_2_O_2_ at room temperature for 15 min to repress the endogenous peroxidase activity. After 1-h 5% bovine serum albumin with 0.05% Triton X-100 blocking, the sections were incubated with primary antibodies overnight at 4 °C, followed by 45-min secondary antibodies incubation at 37 °C. The antibodies of VEGFA (Cat: ab52917, 1:100), CD31 (Cat: ab28364, 1:50), OC2 (Cat: ab28466, 1:100), and EGFL6 (Cat: ab140079, 1:100) were obtained from Abcam. The chromogenic procedures were performed using DAB and hematoxylin reagents. The stained slides were visualized using a bright-field microscope (Olympus) and the positive cells from five random fields were analyzed by ImageJ software.

### Statistical analysis

All the experiments were independently repeated at least three times. The data have been presented as the mean of triplicate values from representative experiments with standard deviation as error bars. The statistical comparison between two groups was analyzed using one-way ANOVA with the least significant difference test. Statistical analysis was performed using SPSS 19.0 software and *p* < 0.05 was considered as statistically significant.

## Results

### Inverse relationship between miR-6086 and the OC2/EGFL6 axis in ovarian cancer cell lines and tissues

**Fig. 1:**
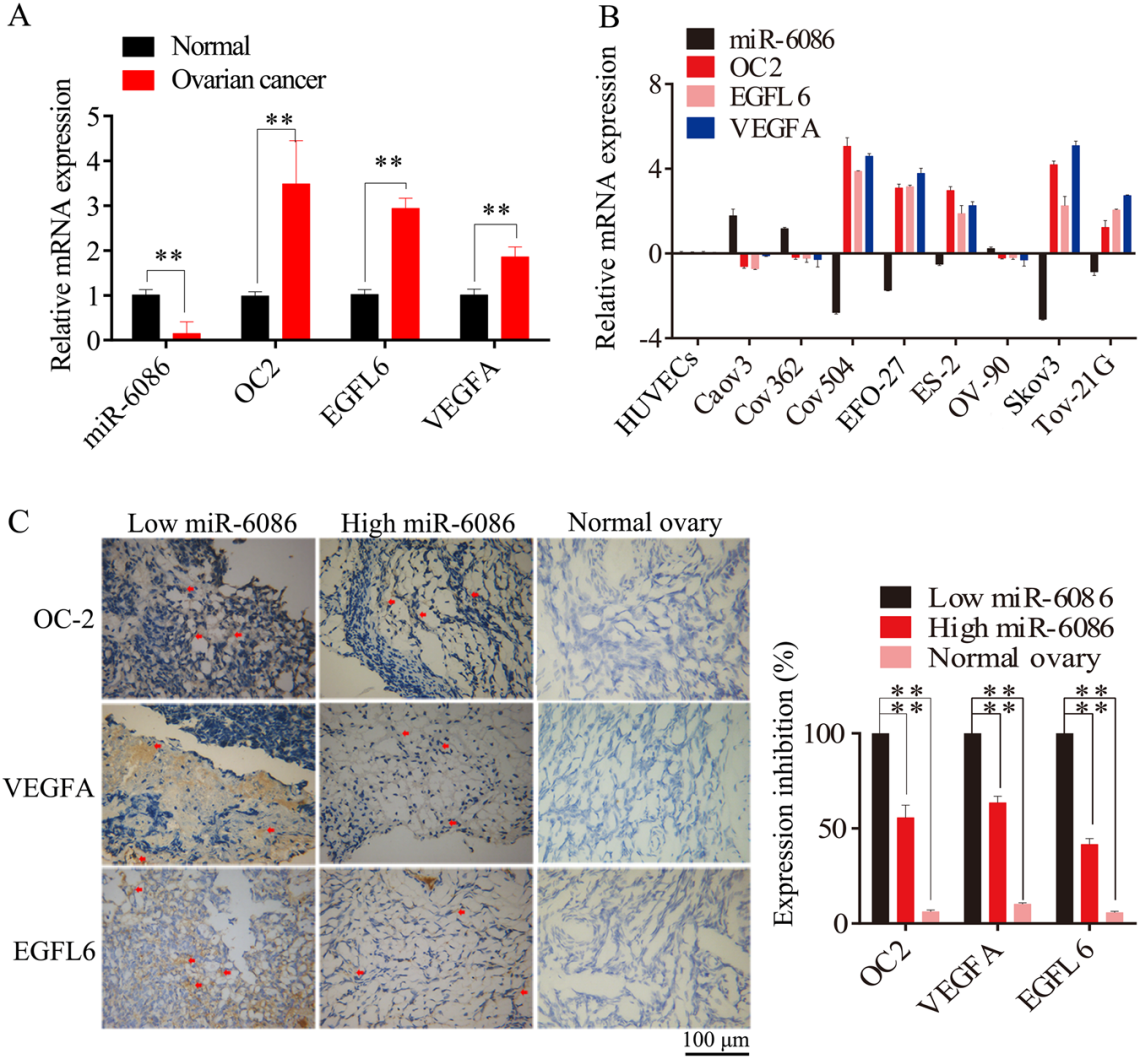
The expression of miR-6086 and OC2/VEGFA/EGFL6 in ovarian cancer cell lines and tissues. **a**, **b** qRT-PCR assays were performed to evaluate the expression levels of miR-6086 and the OC2/VEGFA/EGFL6 axis in ovarian cancer tissues and cell lines. miR-6086 was downregulated and negatively correlated with OC2 (*r* = −0.686, *P* = 0.028), VEGFA (*r* = −0.724, *P* = 0.018), EGFL6 (*r* = −0.790, *P* = 0.007) in vivo (**a** vs. normal ovary tissues) and OC2 (*r* = −0.690, *P* = 0.040), VEGFA (*r* = −0.7203, *P* = 0.029), EGFL6 (*r* = −0.731, *P* = 0.025) in vitro (**b** vs. HUVECs). **c** IHC analysis of the OC2/VEGFA/EGFL6 axis in ovarian cancer tissues with different levels of miR-6086. The spots were calculated by ImageJ software and statistically analyzed in five random fields; scale bars: 100 μm. Data are shown as mean ± SD of three independent experiments. *P < 0.05; **P < 0.01.

We investigated the expression of miR-6086 in the malignant ovary tissue samples (*n* = 33), normal (*n* = 6) ovary tissue samples, ovarian cancer cell lines (*n* = 8), and normal HUVECs, by performing qRT-PCR and IHC assays. The analysis found that 27 of 33 tumor tissues showed a loss of miR-6086 expression compared with the normal tissues, including 10 cases of malignant adenocarcinoma, 7 cases of mucinous carcinoma, 6 cases of epithelial carcinoma, and 4 cases of mixed tumor (Fig. 1a, c). The expression of miR-6086 was down-regulated by 2.79-, 1.73-, 0.51-, 3.11-, 0.87-fold in Cov504, EFO-27, ES-2, Skov3, Tov-21G, and upregulated in Caov3, COV362, and OV-90 compared with HUVECs. Moreover, the average expression of miR-6086 was significantly lower in the ovarian cancer cells than that of the control (Fig. 1b).

Next, our bioinformatics analysis predicted that OC2 and EGFL6 were the direct targets of miR-6086^27–29^. Interestingly, the average expression of OC2 and EGFL6 was significantly higher in ovarian cancer cell lines and tissues than the normal samples and these two factors were observed in obviously positive correlation (Fig. 1a–c). Therefore, there was an inverse relationship between miR-6086 and the OC2/EGFL6 axis both in ovarian cancer cell lines and tissues. To investigate the role of miR-6086 in ovarian cancer, we selected Skov3 with low miR-6086 and high expression of the OC2/EGFL6 axis for subsequent experiments, while Caov3 served as a control.

### Overexpression of miR-6086 negatively regulated tumorigenesis and angiogenesis in ovarian cancer

**Fig. 2:**
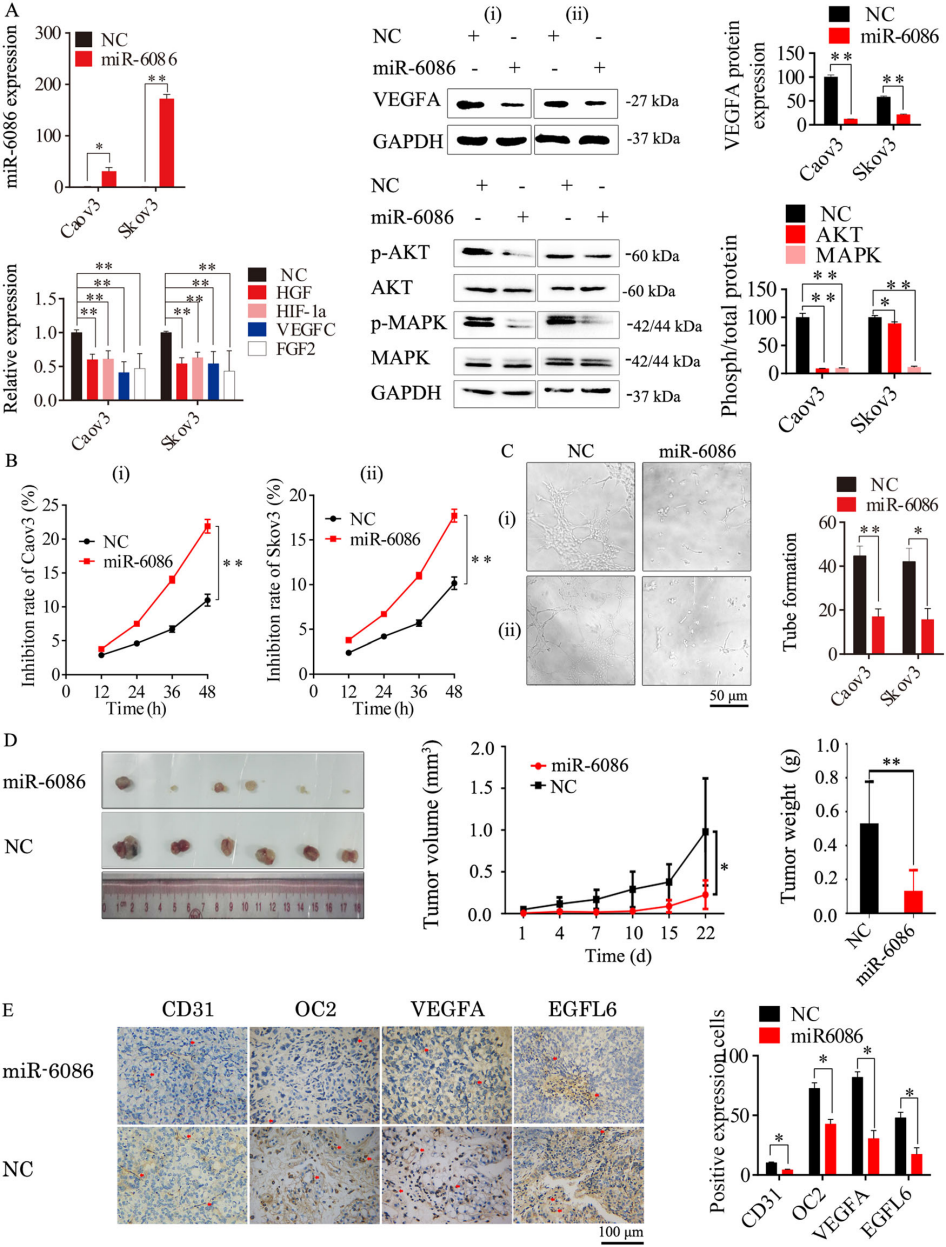
Inhibitory effects of miR-6086 upregulation on tumor growth and angiogenesis. **a** Overexpression of miR-6086 inhibited the expression of angiogenic factors and the activation of AKT/MAPK pathways in Caov3 and Skov3, followed by qRT-PCR and Western blotting assays. **b** CCK-8 assay showed that miR-6086 over-expression inhibited Caov3 and Skov3 proliferation. **c** Representative images and quantitation of HUVECs tube formation. **d** Effect of miR-6086 overexpression on transplanted tumor growth from Skov3, the stripped tumors, tumor size, and tumor weight as indicated (*n* = 6). **e** Representative images and quantitation of the microvessels and the OC2/VEGFA/EGFL6 axis in the stripped tumors, followed by IHC assay. The tubes (**c**) and spots (**e**) were calculated by ImageJ software and statistically analyzed in five random fields; scale bars: 50 μm (**c**), 100 μm (**e**). Data are shown as mean ± SD of three independent experiments. *P < 0.05; **P < 0.01. (i) Caov3, (ii) Skov3.

**Fig. 3:**
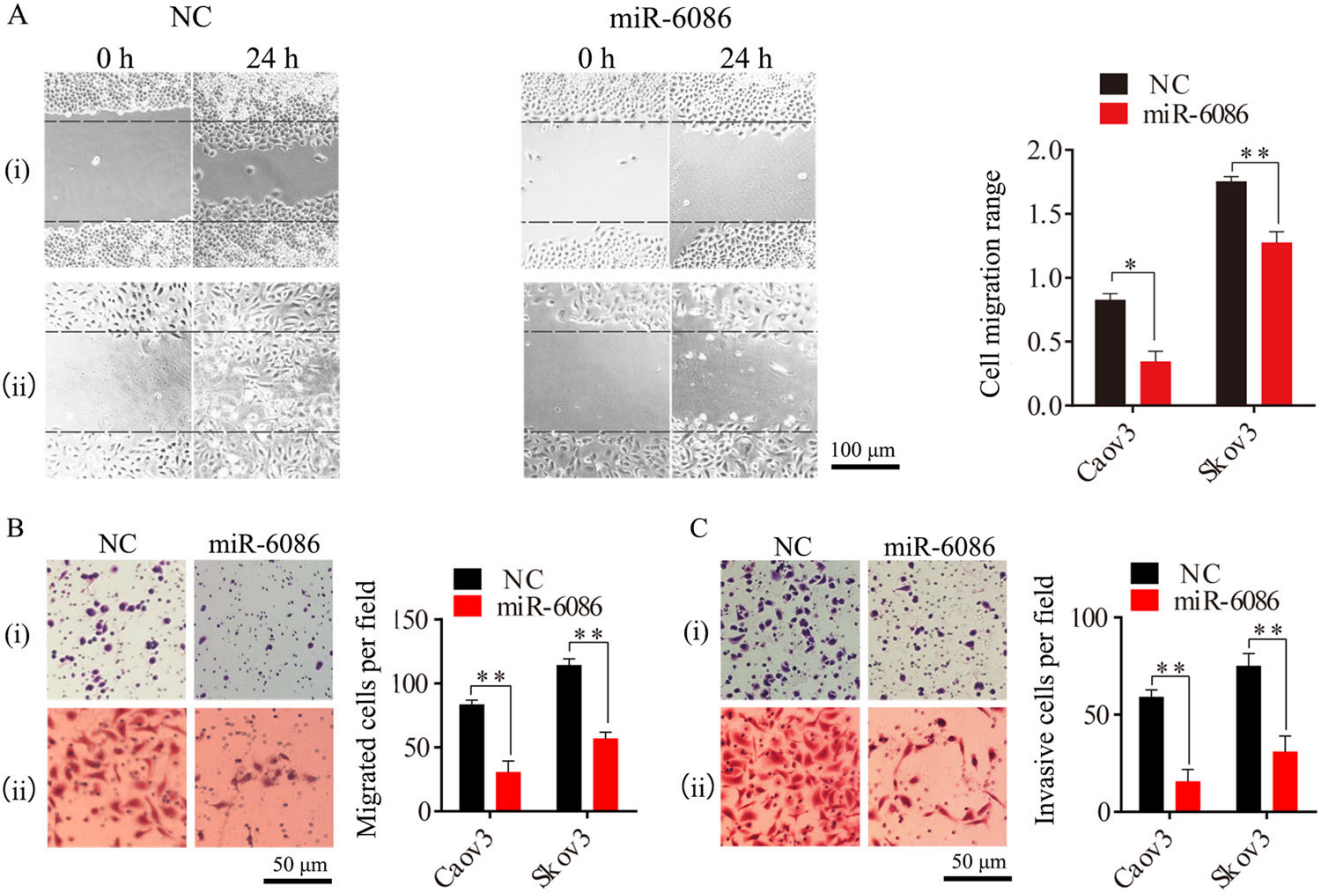
Inhibitory effects of miR-6086 up-regulation on the migration and invasion of Caov3 and Skov3. **a**–**c** Representative images and quantitation of the scratched areas, cell migration, and invasion, followed by wound-healing (**a**), Transwell migration (**b**), and Matrigel invasion (**c**) assays. The scratched areas and migrated cells were calculated by ImageJ software and statistically analyzed in five random fields; scale bars: 100 μm (**a**), 50 μm (**b**, **c**). Data are shown as mean ± SD of three independent experiments. *P < 0.05; **P < 0.01. (i) Caov3, (ii) Skov3.

The expression of miR-6086 was over-expressed in Caov3 and Skov3 with the recombinant plasmid pGCMV-EGFP-miR-6086 and the levels were increased by 30.77- and 172.00-fold (Fig. 2a), leading to a significant decrease of over 70% in the expression of OC2 and EGFL6 (Fig. 5b, e). Moreover, the expression of VEGFA was reduced by over 70% and other angiogenic factors, including hepatocyte growth factor (HGF), hypoxia-inducible factor 1 alpha (HIF-1α), VEGFC, and FGF2, were significantly decreased by 43–63% in these cells (Fig. 2a). Next, we performed the cell proliferation, tube formation, wound-healing, Transwell and tumor challenge experiments with these cells. The results showed that when the expression of miR-6086 was upregulated, the proliferation of Caov3 and Skov3 were significantly repressed at 48 h, with the inhibition by 17.72–21.89% (Fig. 2b). HUVECs were treated by the supernatants from the cells above and the tube number were reduced by more than 60% (Fig. 2c). The migration in the wound-healing assay were significantly decreased by 26.59–59.04% (Fig. 3a) and the migration and invasion inhibition rates in the Transwell assays were exceeded 50% (Fig. 3b, c). In the Skov3-bearing mice, miR-6086 could significantly repress tumor growth, with the inhibition by 77.07% (Fig. 2d). The microvessel density (MVD) was 4.333 ± 0.58 compared with 10.33 ± 0.58 in the control (Fig. 2e). Taken together, the results indicated that overexpression of miR-6086 could negatively regulate ovarian cancer development, by inhibiting tumor growth, migration, invasion, and angiogenesis.

### miR-6086 functioned by decreasing the AKT/MAPK pathways

The AKT/MAPK pathways were activated in numerous cellular events of tumor and endothelial cells including proliferation, migration, and differentiation, with miRNAs functioning as their key regulators^30–32^. Western blotting analysis showed that the phosphorylation of AKT and MAPK were decreased by 91.22% and 93.23% in Caov3, while the levels were decreased by 30.80% and 88.57% in Skov3, respectively (Fig. 2a). These results indicated that miR-6086 negatively regulated the AKT/MAPK pathways to differential extents and suppressed tumorigenesis and angiogenesis in ovarian cancer.

### Depletion of miR-6086 activated tumor angiogenesis

**Fig. 4:**
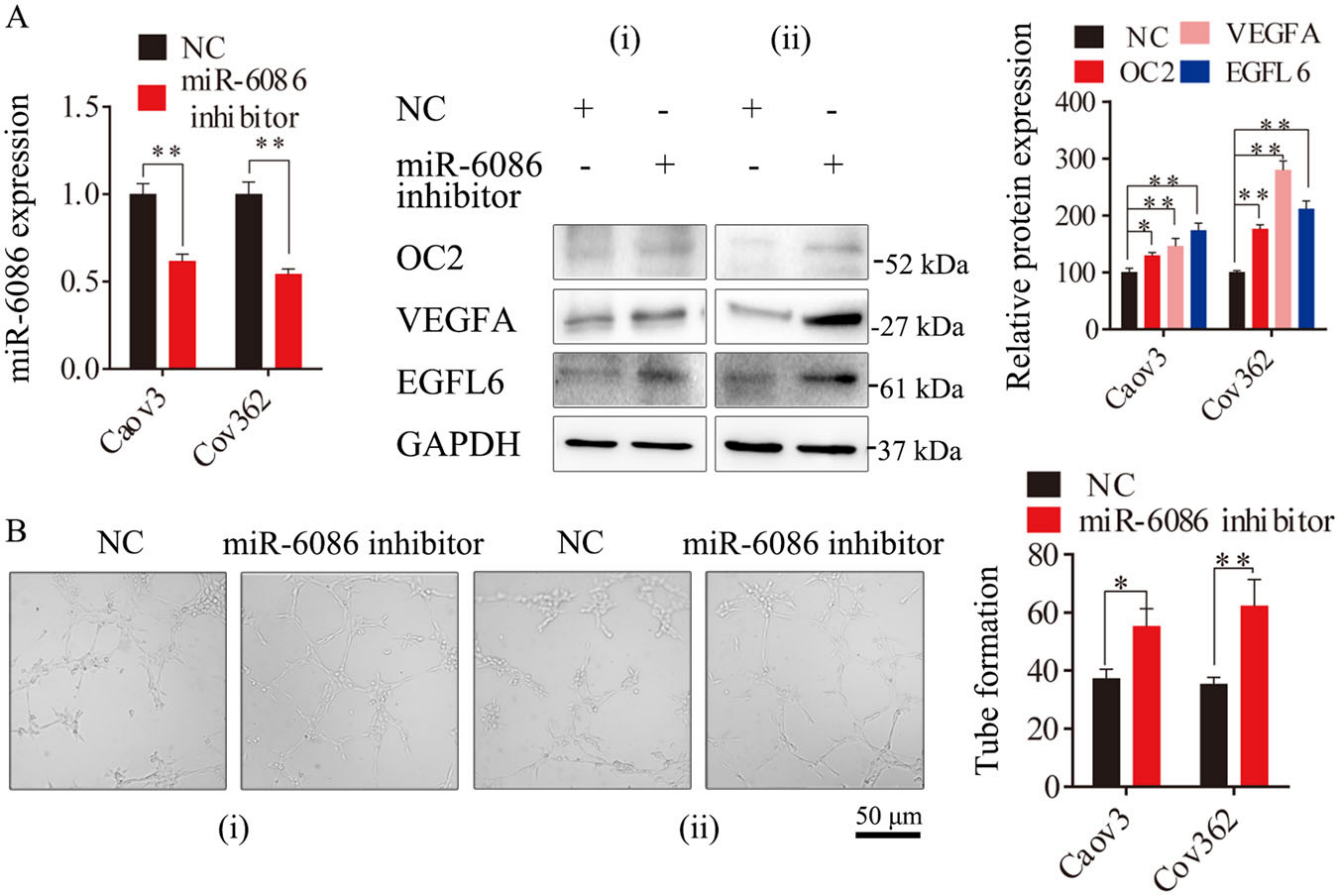
Enhanced effects of miR-6086 inhibitor on ovarian cancer angiogenesis. **a** qRT-PCR and Western blotting assays showed that the expression of the OC2/VEGFA/EGFL6 axis were increased in Caov3 and Cov362 when miR-6086 was silenced by its inhibitor. **b** Representative images and quantitation of HUVECs tube formation; scale bars: 50 μm. Data are shown as mean ± SD of three independent experiments. *P < 0.05; **P < 0.01. (i) Caov3, (ii) Cov362.

miR-6086 were highly expressed in Caov3 and Cov362 (Fig. 1b). Next, in a reciprocal experiment, an inhibitor of miR-6086 was used to deplete its expression in these cells, with a decrease of over 40% (Fig. 4a). In Caov3, the expression of OC2, VEGFA, and EGFL6 were enhanced by 12.84%, 26.33%, 28.19%, respectively, and the tube number treated with cellular supernatant was increased by 32.53%. Similarly, the protein levels were enhanced by 17.39%, 23.18%, 22.19%, and the tube number was increased by 43.32% in Cov362 (Fig. 4b). Our analysis suggested that inhibiting miR-6086 remarkably increased the expression of OC2, VEGFA, and EGFL6, along with an activation in tube formation of HUVECs (Fig. 4b). Although we observed the inhibitory effects of miR-6086 on ovarian cancer progression and angiogenesis, the downstream direct target and molecular mechanism involved were still largely elusive.

### miR-6086 directly downregulated OC_2_ and EGFL6 genes

**Fig. 5:**
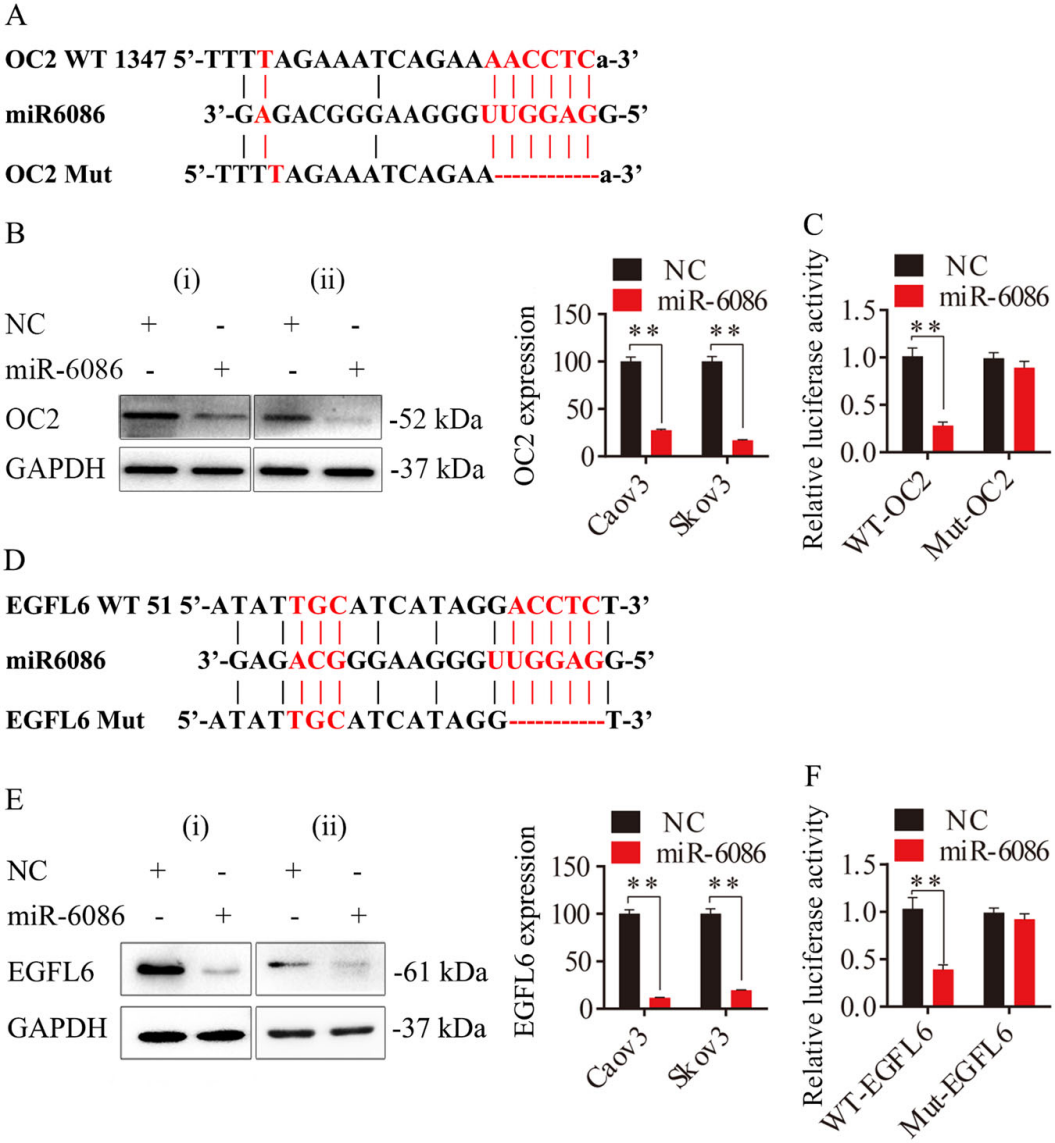
miR-6086 targeted OC2 and EGFL6 and suppressed their expression in ovarian cancer. **a**, **d** Schematic representation of potential binding sites of miR-6086 on 3′UTRs of OC2 (**a**) and EGFL6 (**d**) and the mutant sites. **b**, **e** Protein expression of OC2 (**b**) and EGFL6 (**e**) after treated with miR-6086 in Caov3 and Skov3, followed by Western blotting assay. **c**, **f** The effects of miR-6086 overexpression on the relative luciferase activity of plasmids containing WT or Mut 3′UTRs of OC2 (**c**) and EGFL6 (**f**) mRNAs. Data are shown as mean ± SD of three independent experiments. *P < 0.05; **P < 0.01. (i) Caov3, (ii) Skov3.

miRNAs functioned by binding to the 3′UTR of coding sequence, but this process would not happen if the 3′UTR was mutated (Fig. 5a, d). When miR-6086 was overexpressed, the protein levels of OC2 and EGFL6 were significantly reduced by over 70% (Fig. 5b, e). In contrast, they were approximately increased by 29.71–111.39% with miR-6086 inhibitor (Fig. 4b). The luciferase assay was performed to confirm the bindings of miR-6086 to the 3′UTRs of OC2 and EGFL6. Co-transfection of luciferase plasmids and miR-6086 showed a significant decrease of over 60% in the luciferase activity compared with WT constructs, however, not in the Mut constructs (Fig. 5c, f). Therefore, these results indicated that miR-6086 selectively associated with the 3′UTRs of OC2 and EGFL6 and downregulated their expression.

### Knockdown of OC2 inhibited tumorigenesis and angiogenesis in ovarian cancer

**Fig. 6:**
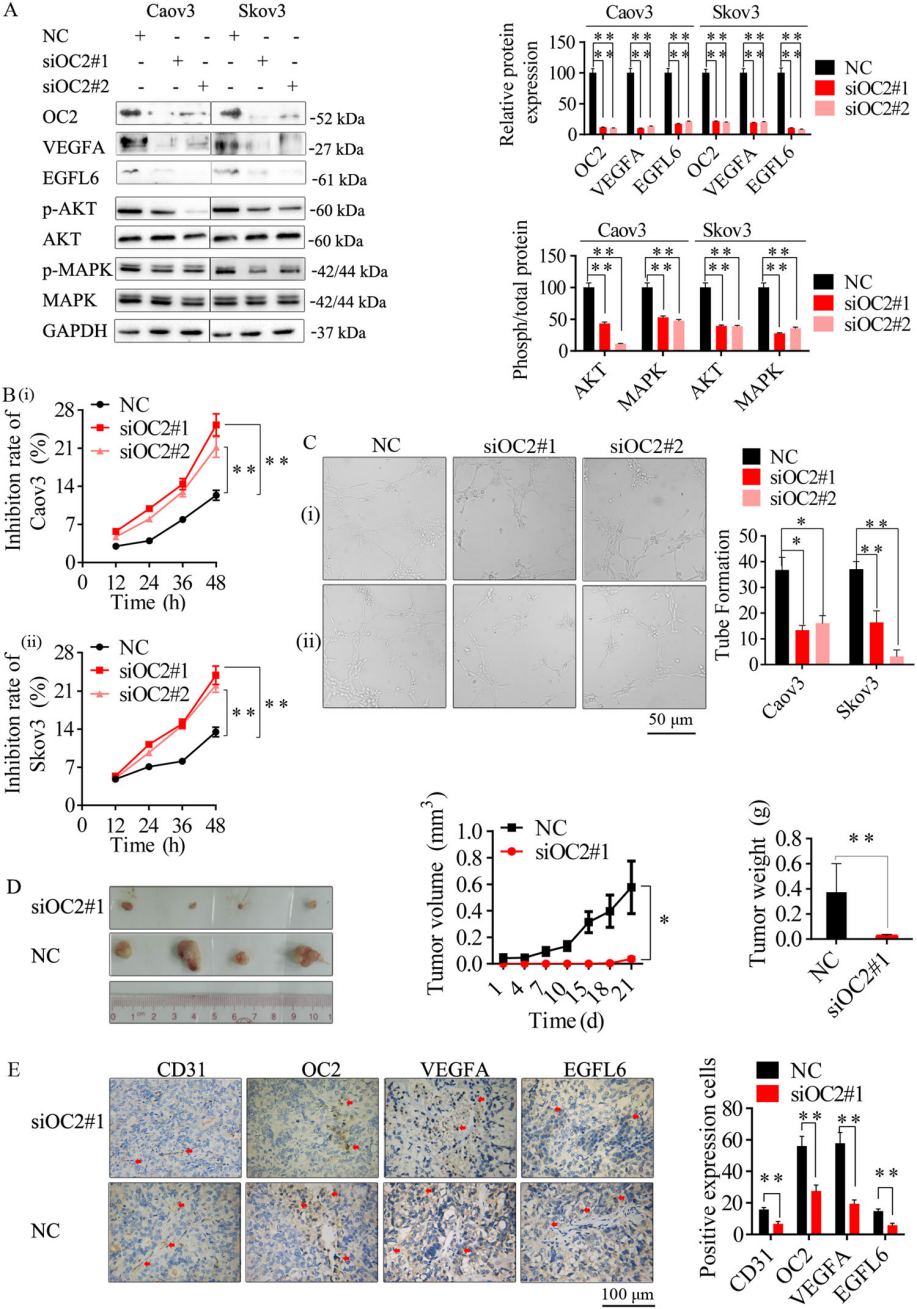
Inhibitory effects of OC2 knockdown on tumor growth and angiogenesis. **a** OC2 knockdown reduced the protein levels of the VEGFA/EGFL6 axis and the AKT/MAPK pathways in Caov3 and Skov3, followed by Western blotting assay. **b** CCK-8 assay showed that OC2 knockdown could inhibit Caov3 and Skov3 proliferation. **c** Representative images and quantitation of HUVECs tube formation. **d** Effect of OC2 knockdown on xenograft tumor growth from Skov3, the stripped tumors, tumor size and tumor weight as indicated (*n* = 4). **e** Representative images and quantitation of the microvessels and the OC2/VEGFA/EGFL6 axis in stripped tumors, followed by IHC assay. The tubes (**c**) and spots (**e**) were calculated by ImageJ software and statistically analyzed in five random fields; scale bars: 50 μm (**c**), 100 μm (**e**). Data are shown as mean ± SD of three independent experiments. *P < 0.05; **P < 0.01. (i) Caov3, (ii) Skov3.

OC2 was identified as one of the direct targets of miR-6086. To further investigate the role of OC2 in ovarian cancer progression and angiogenesis, we decreased its expression by over 70% in Caov3 and Skov3 with the recombinant plasmids pGPU6-GFP-Neo-siOC2#1/siOC2#2. Consistent with our hypothesis, the levels of the VEGFA/EGFL6 axis and the AKT/MAPK pathways were obviously downregulated (Fig. 6a). Further, the cell proliferation were significantly suppressed, with the inhibition rate of 22.09–25.28% (Fig. 6b). The tube formation treated with the supernatants from the cells above were reduced by more than 55% (Fig. 6c). The migration in wound-healing assay were significantly decreased, with the inhibition by 36.90–63.94% (Supplementary Fig. 1a). The migration and invasion inhibition rates in Transwell assays were exceeded 70% (Supplementary Fig. 1b, c). In the Skov3-bearing mice, OC2 knockdown could significantly inhibit the tumor growth by 69.89% (Fig. 6d). The MVD was approximately 7.33 ± 1.04 in the siOC2 mice compared with 18.67 ± 1.29 in the control (Fig. 6e). Taken together, these results indicated that the tumorigenesis and angiogenesis in ovarian cancer could be associated with the pathway downstream to OC2, and this process could be directly regulated by miR-6086.

### Knockdown of EGFL6 inhibited tumorigenesis and angiogenesis in ovarian cancer

**Fig. 7:**
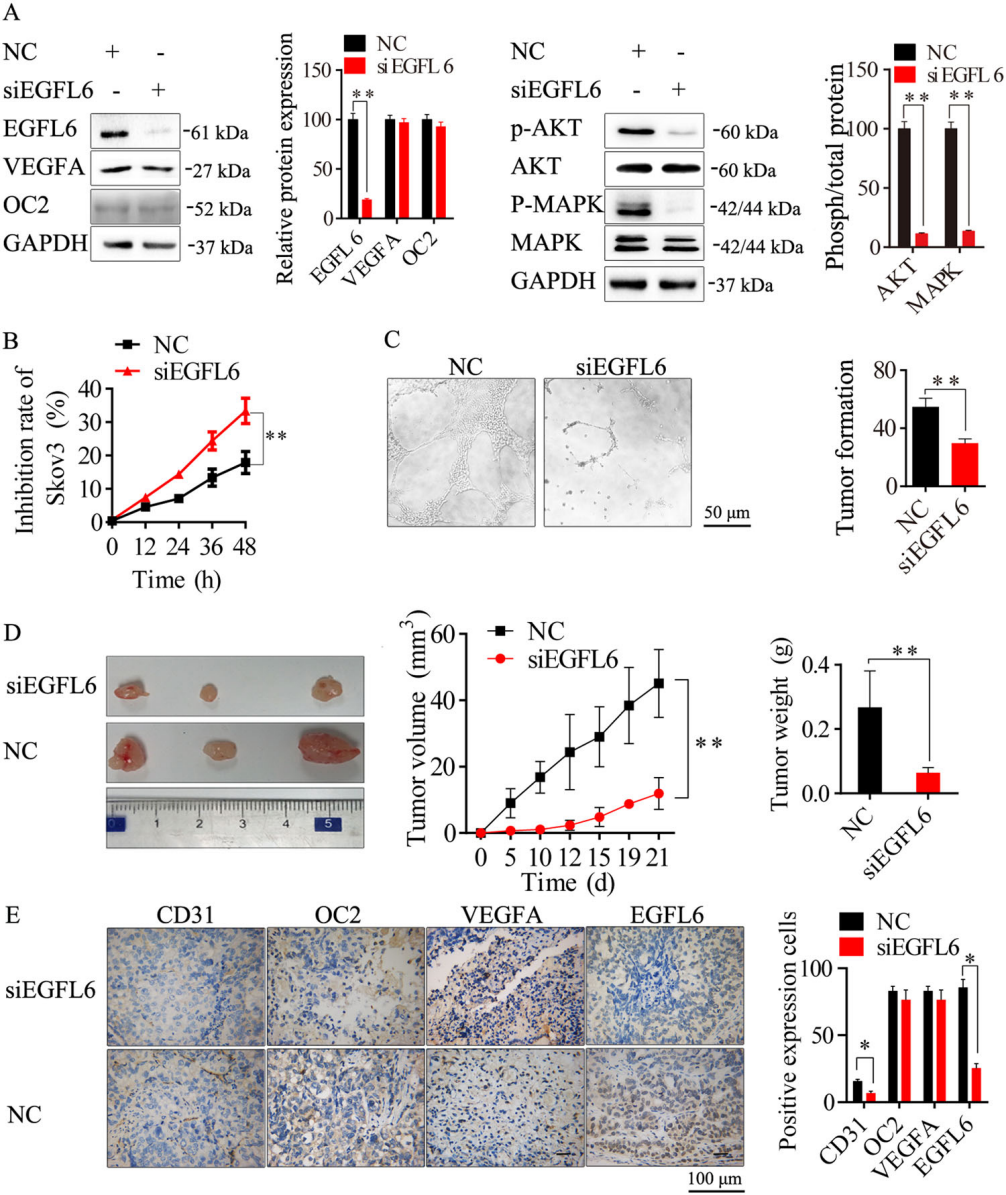
Inhibitory effects of EGFL6 knockdown on tumor growth and angiogenesis. **a** EGFL6 knockdown inhibited the activation of the AKT/MAPK pathways but did not affected the OC2/VEGFA axis in Skov3, followed by Western blotting assay. **b** CCK-8 assay showed that EGFL6 knockdown inhibited Skov3 proliferation. **c** Representative images and quantitation of HUVECs tube formation. **d** Effect of EGFL6 knockdown on transplanted tumor growth from Skov3, the stripped tumors, tumor size, and tumor weight as indicated (*n* = 3). **e** Representative images and quantitation of the microvessels and the OC2/VEGFA/EGFL6 axis of stripped tumors, followed by IHC assay. The tubes (**c**) and spots (**e**) were calculated by ImageJ software and statistically analyzed in five random fields; scale bars: 50 μm (**c**), 100 μm (**e**). Data are shown as mean ± SD of three independent experiments. *P < 0.05; **P < 0.01.

EGFL6 was identified as another direct target of miR-6086. Similarly, to investigate the role of EGFL6 in ovarian cancer progression and angiogenesis, we decreased its expression by over 70% in Skov3 with the recombinant plasmid pGPU6-GFP-Neo-siEGFL6. Our analysis showed that repression of EGFL6 led to an obvious decrease in the p-AKT/p-MAPK levels (Fig. 7a). Moreover, we observed a decrease of 49.21%, 40.31%, and over 60% in the cell proliferation (Fig. 7b), migration (Supplementary Fig. 2a) and invasion (Supplementary Fig. 2b, c) assays, respectively. In addition, there was a significant reduction of 45.73% in tube formation (Fig. 7c). In the animal study, the tumor growth was significantly suppressed by siEGFL6, with the inhibition by 73.62% (Fig. 7d). The MVD was about 6.67 ± 1.14 in the siEGFL6 mice compared with 15.67 ± 2.77 in the control (Fig. 7e). Therefore, the inhibition of EGFL6 could suppressed tumorigenesis and angiogenesis in ovarian cancer. Also this process could be directly regulated by miR-6086.

### The relationship between OC2 and VEGFA/EGFL6 in ovarian cancer

**Fig. 8:**
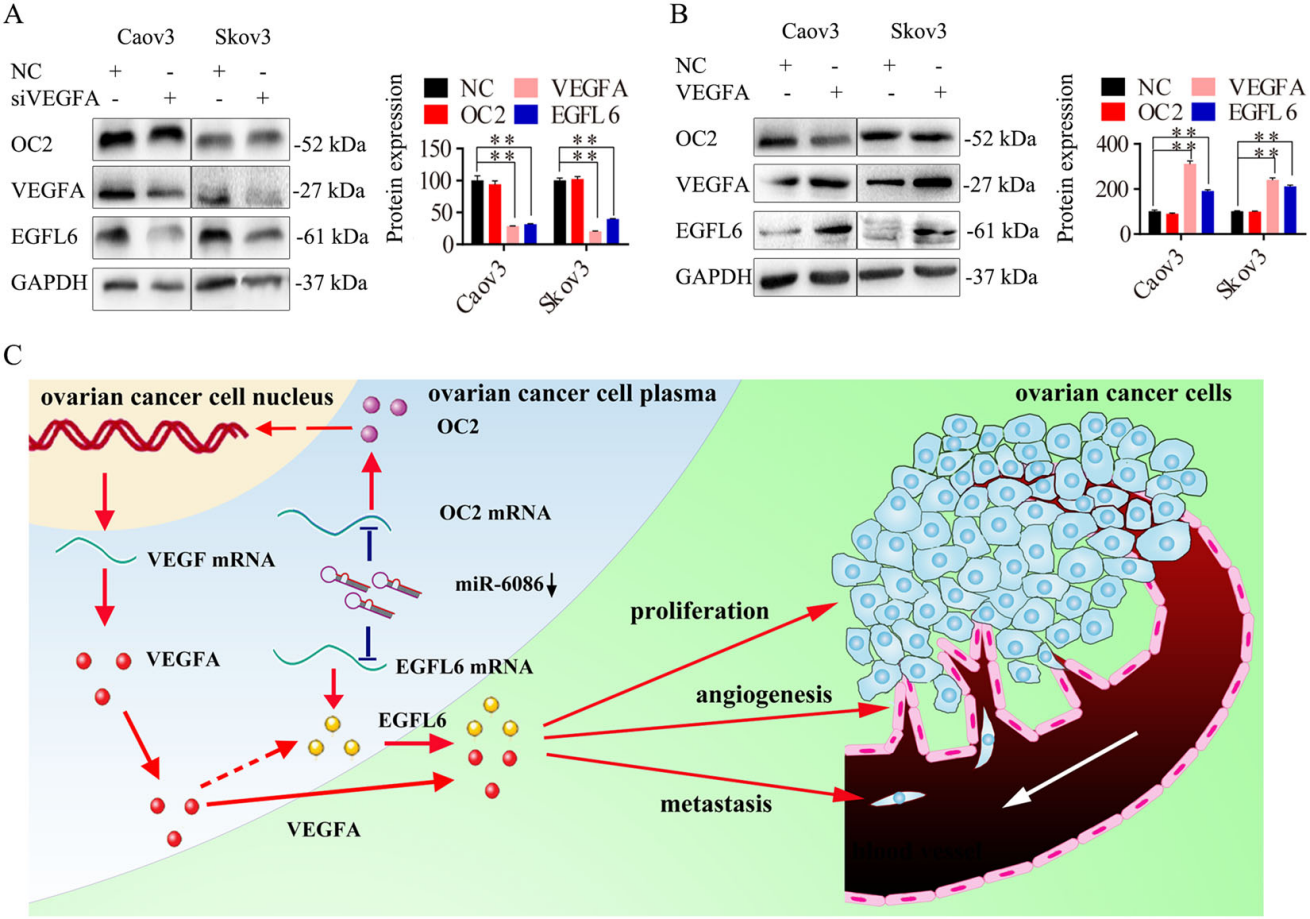
The molecular mechanism of miR-6086 downregulating the OC2/VEGFA/EGFL6 axis in ovarian cancer. **a**, **b** VEGFA was knockdown (**a**) or over-expressed (**b**) in Caov3 and Skov3, followed by Western blotting analysis of the OC2/VEGFA/EGFL6 axis. **c** Schematic representation of miR-6086 molecular mechanism. miR-6086 negatively regulated tumor proliferation, metastasis and angiogenesis in ovarian cancer by down-regulating the OC2/VEGFA/EGFL6 axis in a direct or indirect manner.

The VEGF family, especially VEGFA, is known for the pro-angiogenic activity to mediate downstream signal transduction, exerting the strongest angiogenic effects^33^. Here, the expression of VEGFA was upregulated in ovarian cancer cell lines and tissues (Fig. 1a–c). However, its expression was reduced by over 70% upon the overexpression of miR-6086 in Caov3 and Skov3 (Fig. 2a), while its protein levels were restored by 1.94- and 2.79-fold upon the inhibition of miR-6086 in Caov3 and Cov362 (Fig. 4a). Although the expression of miR-6086 and VEGFA were inversely related, the bioinformatics analysis did not predict association of miR-6086 with the 3′UTR of VEGFA, suggesting an indirect regulation. Since there was a positive correlation between the OC2/EGFL6 axis and VEGFA, we focused on the relationship among OC2, VEGFA, and EGFL6 (Figs. 1a–c, 2e, 4a, and 6a, e). Further, our analysis showed that inhibition of OC2 in Caov3 and Skov3 downregulated the expression of VEGFA, while neither the overexpression nor suppression of VEGFA could affect OC2 expression (Figs. 6a, e and 8a, b). According to the JASPAR database^34^, we observed a binding site of OC2 in the promoter region of VEGFA, suggesting that miR-6086 could regulate VEGFA indirectly via OC2 (Supplementary Table 5). Next, the repression of EGFL6 did not affect the expression of OC2 and VEGFA in Skov3 (Fig. 7a, e). In contrast, the expression of EGFL6 was decreased in Caov3 and Skov3 transfected with siOC2 (Fig. 6a). Moreover, we could not find a binding site of OC2 in the promoter region of EGFL6. However, the expression of EGFL6 was significantly decreased by 18.65–56.72% upon repression of VEGFA and the levels were restored by 2.07 and 2.01-fold upon overexpression of VEGFA (Fig. 8a, b). Thus miR-6086 and OC2 could indirectly regulate EGFL6 expression through VEGFA. Taken together, these results indicated that a tight regulatory network of the OC2/VEGFA/EGFL6 axis functioned downstream to miR-6086 in ovarian cancer.

## Discussion

The miRNAs play a multifaceted role in several solid tumors and fluctuations in their expression may cause dramatic changes in the downstream gene expression pathways and the outcome of cancer cells. Here, we identified a low expression of miR-6086 in the ovarian cancer cells and tissues. Moreover, our functional analysis identified miR-6086 as a master regulator of tumorigenesis and angiogenesis in ovarian cancer by downregulating the OC2/VEGFA/EGFL6 axis. According to luciferase assay, OC2, a member of ONECUT homeodomain transcription factor family, was identified as a direct target of miR-6086. This transcription factor is upregulated during various organ development and cellular events such as differentiation of Th cells in the thymus and insulin secretion in the pancreas, while it is aberrantly expressed in tumor development^35–39^. This is the case in prostate cancer, in which OC2 has been shown to regulate the activity of androgen receptor^40^. In addition, some miRNAs are also related to the regulation of OC2 in tumor cells. miR-9 is frequently down-regulated in primary hepatocellular carcinoma and its restoration retards cell proliferation and migration by targeting IL-6, AP3B1, TC10, OC2, IGF2BP1, MYO1D, and ANXA2^41^. miR-429 inhibits tumor growth and regulates EMT-related marker genes by targeting OC2 in colorectal carcinoma^42^. However, there are only scanty reports stating miRNAs regulate OC2 in tumor angiogenesis. We demonstrated an association of OC2 with the pathway downstream to miR-6086, but their roles in ovarian cancer remain to be understood in details.

EGFL6, another direct target of miR-6086, contains homologous EGF structural domains, a MAM domain and an integrin binding site (Arg-Gly-Asp, RGD) and is predominantly released during early developmental stages^43^. It shows high expression in tumor-associated endothelial cells than normal ovarian and wound-associated endothelial cells, which could trigger endothelial cell proliferation, migration and angiogenesis through activating the AKT/MAPK pathway^44–47^. Indeed, EGFL6 regulates the asymmetric division, maintenance and metastasis of ovarian cancer stem-like cells^6^. We demonstrated another association of EGFL6 with the pathway downstream to miR-6086, which may serve as a unique biomarker for ovarian cancer therapy.

In this study, suppressing the expression of OC2 or EGFL6 could inhibit tumorigenesis, angiogenesis and relevant factor secretion in ovarian cancer. We observed an intricate association of the OC2/VEGFA/EGFL6 axis which could promote angiogenesis and tumorigenesis in ovarian cancer, together with other angiogenic factors. We demonstrated that OC2 could directly up-regulate VEGFA and indirectly upregulate EGFL6 through VEGFA but altering the expression of EGFL6 did not affect the protein levels of OC2 or VEGFA. Our findings identified miR-6086 as a direct negative regulator of the OC2/EGFL6 pathways and indirectly regulate VEGFA via OC2. In addition, other angiogenic factors such as HGF, HIF-1α, VEGFC, and FGF2 were downregulated to varying degrees upon over-expression of miR-6086 (Fig. 2a)^48^. Interestingly, miR-6086 has binding sites in the 3′UTR of HGF and HIF-1α and we also found binding sites of OC2 in the promoter regions of VEGFC and FGF2. Therefore, miR-6086 may act as a master negative regulator to suppress the angiogenesis networks of ovarian cancer in a direct or indirect manner. However, further investigations are necessary to ascertain the exact relationship between miR-6086, OC2, and these angiogenic factors, together with identification of additional miRNA-target gene networks regulating angiogenesis in ovarian cancer.

In conclusion, we have elucidated the tight regulatory network of miR-6086 that interacts with the OC2/VEGFA/EGFL6 axis and our study reveals a promising insight into understanding their roles of tumorigenesis and angiogenesis in ovarian cancer, ultimately providing potential molecular targets for anti-tumor therapeutics. However, the effects of over-expression of miR-6086 in animal models will validate our findings in vitro, which may provide intricate details on its exact role in ovarian cancer. Moreover, the effective miRNA-delivery systems are necessary for such approaches to be successful^1^.

## Supplementary information

Supplemental materials

Supplementary Fig. 1

Supplementary Fig. 2
