# Supplementary material for: miR-6086 inhibits ovarian cancer angiogenesis by downregulating the OC2/VEGFA/EGFL6 axis
Source: Cell Death Dis. 2020 May 11;11(5):345. doi: 10.1038/s41419-020-2501-5 (PMC7214437; doi:10.1038/s41419-020-2501-5)
Supplement: Supplementary file 1 — Supplemental materials [file 41419_2020_2501_MOESM1_ESM.doc]

**Supplementary materials for**

**miR-6086 inhibits ovarian cancer angiogenesis by down-regulating the OC2/VEGFA/EGFL6 axis**

**Supplementary** **Fig. 1 Inhibitory effects of OC2 knockdown on the migration and invasion of Caov3 and Skov3.** **a**-**c** Representative images and quantitation of the scratched areas, cell migration and invasion, followed by wound-healing (**a**), Transwell migration (**b**) and Matrigel invasion (**c**) assays. The scratched areas and migrated cells were calculated by ImageJ software and statistically analyzed in five random fields; scale bars: 100 μm (**a**), 50 μm (**b**, **c**). Data are shown as mean±SD of three independent experiments. **P* <0.05; ***P* <0.01. (i) Caov3, (ii) Skov3.

**Supplementary** **Fig. 2 Inhibitory effects of EGFL6 knockdown on the migration and invasion of Skov3. a**-**c** Representative images and quantitation of the scratched areas, cell migration and invasion, followed by wound-healing (**a**), Transwell migration (**b**) and Matrigel invasion (**c**) assays. The scratched areas and migrated cells were calculated by ImageJ software and statistically analyzed in five random fields; scale bars: 100 μm (**a**), 50 μm (**b**, **c**). Data are shown as mean±SD of three independent experiments. **P* <0.05; ***P* <0.01.

**Supplementary Table 1. The sequences of miR-6086, siOC2s, siEGFL6 and siVEGFA**

| **Name** | **Sites of ORF** | **Sequences (5’-3’)** |
| --- | --- | --- |
| miR-6086  (MIMAT0023711) | - | 5’-GGAGGUUGGGAAGGGCAGAG-3’ |
| Pre-mR-6086  sense | - | 5’-GAATTCGGAGGTTGGGAAGGGCAGAGGTTTTGGCCACTGACTGACCTCTGCCCCCCAACCTCCACCGGT-3’  (*EcoRI*/*AgeI*) |
| Pre-mR-6086  anti-sense | - | 5’-ACCGGTGGAGGTTGGGGGGCAGAGGTCAGTCAGTGGCCAAAACCTCTGCCCTTCCCAACCTCCGAATTC-3’  (*EcoRI*/*AgeI*) |
| NC miRNA | - | 5’-AAATGTACTGCGCGTGGAGAC-3’ |
| siOC2#1 | 1015-1034 | 5’-GCCAGCTGGAAGAAATCAACA-3’ |
| siOC2#2 | 1277-1296 | 5’-GCAAGAACCAAACAAAGACAG-3’ |
| siEGFL6 | 1700-1719 | 5’-GAGACAAAGUCGGGAAACUTT-3’ |
| siVEGFA | 1227 -1245 | 5’-GGAGTACCCTGATGAGATCTT-3’ |
| NC siRNA | - | 5’-UUCUCCGAACGUGUCACGUTT-3’ |

**Supplementary Table 2. The primers for qRT-PCR**

| **Primers** | **Forward (F) and Reverse (R) Sequences (5’-3’)** |
| --- | --- |
| miR-6086  (reverse transcription) | 5’-CTCAACTGGTGTCGTGGAGTCGGCAATTCAGTTGAGctctgccc-3’ |
| mR-6086 | 5’-TGGTGTCGTGGAGTCG-3’ |
| U6-F | 5’-CTCGCTTCGGCAGCACA-3’ |
| U6-R | 5’-AACGCTTCACGAATTTGCGT-3’ |
| OC2-F | 5’-CCCAAGCTTATGAAGGCTGCCTACACCGCCTAT-3’ |
| OC2-R | 5’-TCCCCGCGGTGCTTTGGTACACGTGCTGGA-3’ |
| EGFL6-F | 5’-CCCAAGCTTATGCCTCTGCCCTGGAGCCTT-3’ |
| EGFL6-R | 5’-TCCCCGCGGTCAGTCATCCACAGATAAAAGG-3’ |
| FGF2-F | 5’-CCAGTTCGATTCAGTGCCACA-3’ |
| FGF2-R | 5’-GTGTGCTAACCGTTACCTGGCTATG-3’ |
| VEGFC-F | 5’-GTGTCCAGTGTAGATGAA-3’ |
| VEGFC-R | 5’-CCTGTTCTCTGTTATGTTG-3’ |
| HGF-F | 5’-ACGCTACGAAGTCTGTGA-3’ |
| HGF-R | 5’-AAGAATTTGTGCCGGTGT-3’ |
| HIF-1α-F | 5’-TCTGGGTTGAAACTCAAGCAACTG-3’ |
| HIF-1α-R | 5’-CAACCGGTTTAAGGACACATTCTG-3’ |
| GAPDH-F | 5’-TCTCTGCTCCTCCTGTTC-3’ |
| GAPDH-R | 5’-TGCTTCACCACCTTCTTG-3’ |

**Supplementary Table 3. The reaction parameters for qRT-PCR**

| **Reaction parameters** | **Volume** |
| --- | --- |
| qRT-PCR mix | 10 μL |
| Forward primer | 0.5 μL |
| Reverse primer | 0.5 μL |
| cDNA | 1 μL |
| ddH_2_O | 13 μL |
| Total | 25 μL |

**Supplementary Table 4. The temperature protocols for qRT-PCR**

| **Protocol** | **Temperature** | **Time** | **Cycle** |
| --- | --- | --- | --- |
| Initial denaturation | 95 ℃ | 2 min | 1 |
| Denaturation | 95 ℃ | 10 s | 40 |
| Primer annealing | 55 ℃ | 30 s |  |
| Extension | 68 ℃ | 30 s |  |
| Melting curve | 72 ℃ | 2 min | 1 |
| Store | -4 ℃ |  |  |

**Supplementary Table 5. The binding affinity of OC2 on the VEGFA promoter region**

| **Matrix ID** | **Name** | **Score** | **Relative score** | **Sequence ID** | **Start** | **End** | **Strand** | **Predicted sequence** |
| --- | --- | --- | --- | --- | --- | --- | --- | --- |
| MA0756.1 | OC2 | 2.24 | 0.7331 | VEGFA | 98 | 111 | + | aggaaataaacatt |
| MA0756.1 | OC2 | 1.36 | 0.7208 | VEGFA | 101 | 114 | + | aaataaacatttac |
