# Supplementary figures and images for: miR-6086 inhibits ovarian cancer angiogenesis by downregulating the OC2/VEGFA/EGFL6 axis
Source: Cell Death Dis. 2020 May 11;11(5):345. doi: 10.1038/s41419-020-2501-5 (PMC7214437; doi:10.1038/s41419-020-2501-5)

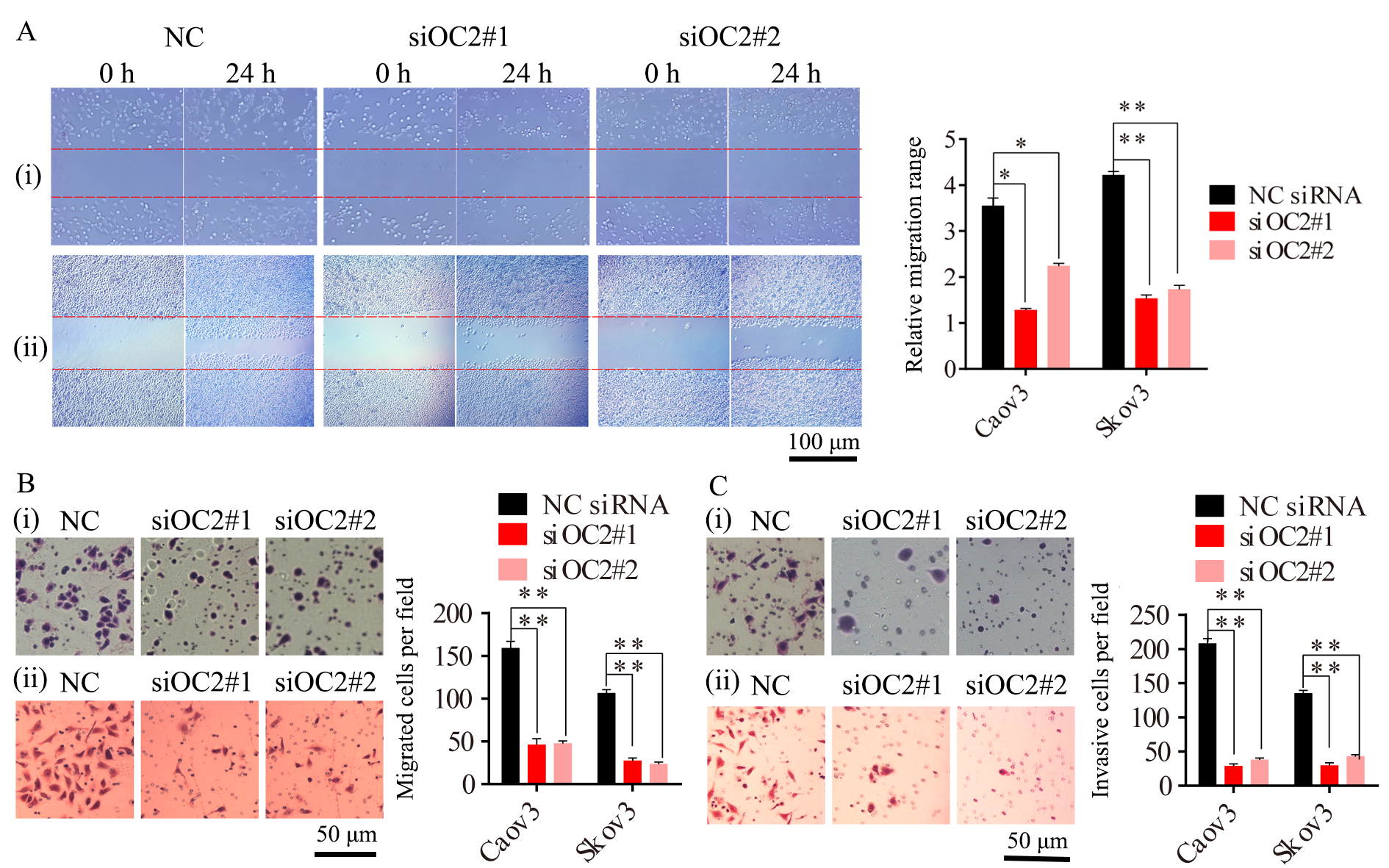

Supplement: Supplementary file 2 — Supplementary Fig. 1 [file 41419_2020_2501_MOESM2_ESM.tif]

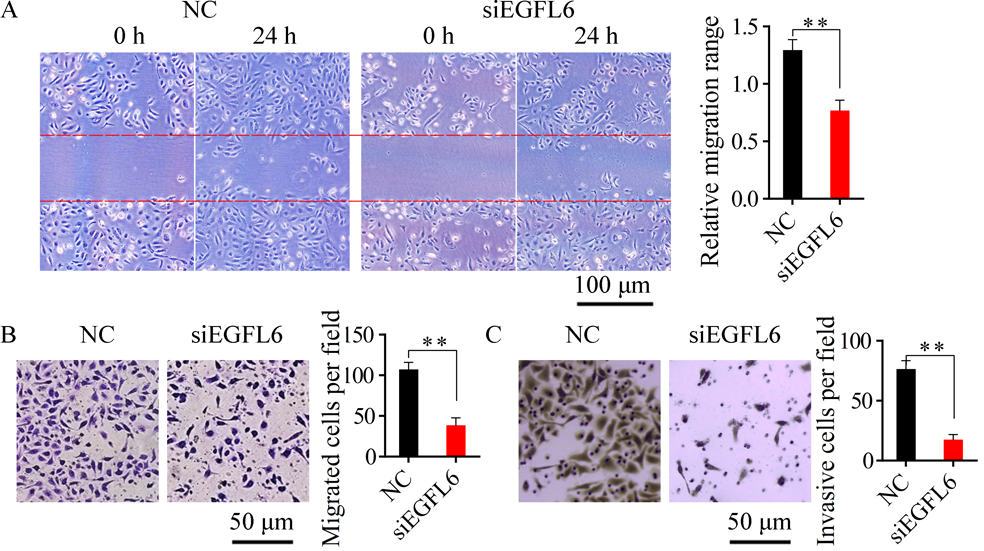

Supplement: Supplementary file 3 — Supplementary Fig. 2 [file 41419_2020_2501_MOESM3_ESM.tif]
